# Quality and Safety Outcomes of a Hospital Merger Following a Full Integration at a Safety Net Hospital

**DOI:** 10.1001/jamanetworkopen.2021.42382

**Published:** 2022-01-06

**Authors:** Erwin Wang, Sonia Arnold, Simon Jones, Yan Zhang, Frank Volpicelli, Joseph Weisstuch, Leora Horwitz, Bret Rudy

**Affiliations:** 1 NYU Langone Health, Brooklyn, New York

## Abstract

**Importance:**

Hospital consolidations have been shown not to improve quality on average.

**Objective:**

To assess a full-integration approach to hospital mergers based on quality metrics in a safety net hospital acquired by an urban academic health system.

**Design, Setting, and Participants:**

This quality improvement study analyzed outcomes for all nonpsychiatric, nonrehabilitation, non-newborn patients discharged between September 1, 2010, and August 31, 2019, at a US safety net hospital that was acquired by an urban academic health system in January 2016. Interrupted time series and statistical process control analyses were used to assess the main outcomes and measures. Data sources included the hospital’s electronic health record, Centers for Medicare & Medicaid Services Hospital Compare, and nursing quality reports.

**Exposures:**

A full-integration approach to the merger that included: (1) early administrative and clinical leadership integration with the academic health system; (2) rapid transition to the academic health system electronic health record; (3) local ownership of quality metrics; (4) system-level goals with real-time actionable analytics through combined dashboards; and (5) implementation of value-based and other analytic-driven interventions.

**Main Outcomes and Measures:**

The primary outcome was in-hospital mortality. Secondary outcomes included 30-day readmission, patient experience, and hospital-acquired conditions.

**Results:**

The 122 348 patients in the premerger (September 2010 through August 2016) and the 58 904 patients in the postmerger (September 2016 through August 2019) periods had a mean (SD) age of 55.5 (22.0) years; the total sample of 181 252 patients included 112 191 women (61.9%), the payor mix was majority governmental (144 375 patients [79.7%]), and most admissions were emergent (121 469 patients [67.0%]). There was a 0.71% (95% CI, 0.57%-0.86%) absolute (27% relative) reduction in the crude mortality rate and 0.95% (95% CI, 0.83%-1.12%) absolute (33% relative) in the adjusted rate by the end of the 3-year intervention period. There was no significant improvement in readmission rates after accounting for baseline trends. There were fewer central line infections per 1000 catheter days, fewer catheter-associated urinary tract infections per 1000 discharges, and a higher likelihood of patients recommending the hospital or ranking it 9 or 10.

**Conclusions and Relevance:**

In this quality improvement study, a hospital merger with a full-integration approach to consolidation was found to be associated with improvement in quality outcomes.

## Key Points

**Question:**

Is a full-integration approach to a hospital merger associated with improved quality in an acquired hospital?

**Findings:**

This quality improvement study of 181 252 patients found that a full-integration approach to a hospital merger was associated with an absolute reduction in crude and adjusted mortality rates by the end of the 3-year intervention period.

**Meaning:**

These results suggest that a full clinical and operational integration approach to a hospital merger may improve outcomes as measured by quality and safety metrics, including mortality rates.

## Teaser

This quality improvement study assesses a full-integration approach to hospital mergers using quality metrics that track hospital mortality and readmission.

## Introduction

The rate of hospital consolidation has more than doubled since 2009.^1^ Evidence suggests an overall negative effect of mergers on quality. Most studies show increased overall mortality following decreased hospital competition.^2,3,4,5^ A more recent study suggests overall mortality and readmission rates do not improve with hospital mergers and patient experience may worsen, even in consolidations with high-quality acquirers.^6^

These disappointing outcomes may be due to decreased incentives for effective management. Previous studies have shown that most consolidations are largely characterized as lacking meaningful integration of management, culture, and data systems and are typically associated with decreased competition, more concentrated markets, and less innovation.^7,8^ The research concludes that this vastly common approach to consolidation suggests financial rather than quality motivations for mergers.

One study suggests that merger strategies that include meaningful integration could improve quality.^9^ As most mergers lack significant operational integration, research is limited in describing the effect of such an approach to hospital consolidation. Our study assessed whether acquisition of a noncompetitor, academic, safety net hospital by an academic health system with a full-integration strategy was associated with improved quality.

## Methods

### Study Setting and Design

NYU Langone Health (NYULH) is an urban academic medical system. Before the merger, NYULH consisted of a multispecialty academic acute care hospital (450 beds) and a specialized orthopedic, rheumatic, and neurologic treatment and rehabilitation hospital (190 beds). Lutheran Medical Center (450 beds) was a teaching hospital located near preexisting NYULH outpatient sites. Most individuals in its catchment area were Medicaid or Medicare beneficiaries or uninsured, representing the densest noncommercial payor communities for nonpublic hospitals in the country. Before the merger, funding was lacking for technology and infrastructure investments to support quality improvement. As a result, there was variation from standard clinical practice, such as sending patients for elective procedures through the emergency department. Outcome metrics (eg, mortality rates) were poor.

In January 2016, NYULH completed a full asset merger with Lutheran, later renamed NYU Langone Hospital–Brooklyn (NYULHB), to add an institution to the system convenient for Brooklyn patients. Unlike most mergers, the goals of this acquisition were not exclusively financial, as NYULH looked to (1) integrate NYULHB fully into the NYULH system and thus improve quality and (2) expand clinical capabilities beyond that of a typical safety net hospital to become more than solely a referral base, thus fulfilling NYULHB’s academic mission. Following the merger, several interventions were made to improve NYULHB’s performance: (1) administrative and clinical leadership were integrated into the NYULH system early in the process; (2) patient records and physician and billing data were transitioned into NYULH electronic health record (EHR) and information systems; (3) accountability for quality metrics was established among local ownership; (4) system-level quality goals identical to those across NYULH were adopted, with real-time performance assessed with actionable analytics through online dashboards; and, (5) value-based, analytic-driven interventions were implemented.

The premerger period was September 1, 2010, through August 31, 2016. The postmerger period was September 1, 2016, through August 31, 2019. The primary outcome metric was in-hospital mortality; secondary outcome metrics were in-hospital readmissions, hospital acquired conditions (HACs) (ie, catheter-associated urinary tract infections [CAUTIs] and central line-associated bloodstream infections [CLABSIs]); and patient experience scores measured through Hospital Consumer Assessment of Healthcare, Providers, and Systems (HCAHPS) surveys. We used 2 approaches to analyze pre- and postmerger outcomes, interrupted time series (ITS) and statistical process control (SPC). This study follows the Standards for Quality Improvement Reporting Excellence (SQUIRE) reporting guideline for quality improvement research. Because this study used a limited data set that could not be linked back to patients, it was not considered human participant research and was exempt from the NYU Grossman School of Medicine institutional review board and informed consent requirements.

### Study Setting

#### Leadership Integration

Administrative and clinical leadership changes were among the first interventions. A leadership governance structure more typical of academic systems was implemented to reinforce accountability and dedication to quality while ensuring local autonomy to manage specific operational challenges. Many physician leaders before the merger held part-time hospital appointments alongside full-time private practice commitments. To enhance accountability, these leaders were replaced by full-time, employed academic physicians. Clinical chiefs had specific quality and operational targets.

Following the merger, new service lines in NYULHB included reconstructive breast surgery, spine surgery, robotic surgery, advanced endoscopy, and advanced bronchoscopy. Contracted services for physician groups in emergency medicine, medical intensive care, and radiology were replaced by full-time employed physicians. All graduate medical education programs were consolidated into the NYU Grossman School of Medicine.

#### Information Technology Transition

Before the merger, NYULHB had a patchwork of record systems. A comprehensive EHR and cost-accounting system was implemented September 2016 that was integrated with NYULH. EHR and cost-accounting data were aggregated in systemwide dashboards with real-time analytics, enabling visualization of clinical and operational performance. The data included patient-level information and inpatient physician and hospital billing from cost-accounting records for accurate, real-time estimation down to case-level details. Expense groupings aligned with clinical intuition (eg, laboratory or pharmacy, rather than administrative, categories) for ease of use for clinical and administrative leaders.

Following installation of integrated information and data systems, existing NYULH quality dashboards were transitioned to NYULHB in December 2016. Metrics were compared with internal benchmarks set using prior and anticipated future performance and against comparable hospitals. Expected outcomes, eg, mortality, were obtained from standardized risk-adjustment modeling algorithms (Vizient Inc) using admissions from hundreds of hospitals.

#### Local Ownership of Quality Outcomes

Premerger data demonstrated opportunity to improve quality. An area of initial focus was instituting and expanding quality committees and infrastructure such as root cause analyses and occurrence review committees. Prior analyses involved part-time, voluntary clinical leaders; postmerger meetings with employed full-time clinical leadership facilitated review of a broader number and breadth of cases and reinforced accountability to address issues affecting quality.

An integrated quality and performance improvement plan for NYULHB and the full system reinforced transparency and accountability while balancing local circumstances with system goals. This plan included targets for quality measures (eg, mortality rates and HACs), with eventual identical quality targets with the system.

#### Value-Based, Analytic-Driven Interventions

Value-based, analytic-driven initiatives from NYULH were introduced at NYULHB.^6^ Projects were transitioned to NYULHB alongside information technology implementation, including EHR-embedded decision support (eg, guideline-based blood transfusions).^7^ Based on poor performance in key areas and trends seen in dashboards, additional projects targeted novel improvement opportunities at NYULHB (eg, HACs).

### Study Design

The primary outcome metric was in-hospital, all-cause mortality. Secondary outcomes were 30-day same hospital readmissions, HACs, and patient experience. HACs (ie, CLABSIs and CAUTIs) were assessed as infections per 1000 device-days and per 1000 discharges.^8^ Rates per catheter-day and per discharge were both included because interventions to reduce infection rates included both avoidance of device placement (ie, fewer patients with any device) and initiatives to reduce risk in those with devices. All events were routinely identified by the quality department following national standards for case finding. Patient experience was assessed using HCAHPS outcomes, which were restricted to responses from NYULHB.

#### Study Cohort and Data Sources

For patient experience measures, we used yearly Centers for Medicare & Medicaid (CMS) Hospital Compare reports—2014 to 2016 for premerger outcomes and 2017 to 2019 for postmerger.^10^ We excluded admissions to rehabilitation, psychiatry, and normal newborn deliveries, which is standard in American Hospital Association and CMS cost accounting. Data sources included the hospital’s EHR, CMS Hospital Compare, and nursing quality reports.

#### Independent Variables

The primary exposure variable was intervention period. To account for potential changes in patient mix over time, mortality and readmission models included the following covariates at the time of admission: age (as indicators for 5-year age bins up to 90 years and an indicator for 91 years and older); sex; race and ethnicity (White, Black, other, or unknown [racial and ethnic categories were limited because of small numbers to those listed to ensure models converged]); insurance (Medicare, Medicaid, self-pay, commercial, or other insurance types); a surgical patient indicator, grouped using MS-DRG (Medicare Severity–Diagnosis Related Group); DRG weight (in log[DRG weights]); Elixhauser comorbidities; and calendar quarter as seasonal indicators.^9^ Only 240 of 174 544 admissions (0.1%) were missing covariate data, and these were excluded from our analysis. We fixed all DRG weights to 2019 weights except for DRG codes that were changed or retired, in which case we took the most recent available weight. The average DRG weight for all hospitalizations represents the case mix index.

### Statistical Analysis

We used descriptive statistics to characterize pre- and postintervention cohort demographics. Categorical variables were summarized as frequencies and proportions, and continuous variables as means. We examined differences in pre- and postintervention cohorts using χ^2^ tests or *t* tests, as appropriate.

For mortality and readmission outcomes, we conducted complementary SPC and ITS analyses to determine whether postintervention outcomes differed from preintervention. First, we plotted quarterly mortality rate for all patients on an SPC chart. We explored whether there was special-cause variation in the postintervention period, defined as any rates above or below the 3-SD (99.7%) control limits or a run of at least 8 consecutive observations above or below the mean.^11^ We constructed SPC charts for HACs and patient experience; there were insufficient data points for ITS analyses for these outcome metrics.

We then conducted ITS analyses for mortality and readmissions. This approach is superior to a simple pre-post analysis because it accounts for underlying preintervention trends. We conducted sequential ITS analyses, first without adjustment, then adjusted for all covariates but log DRG weight (which could potentially be influenced by changes in coding over time), and then fully adjusted. In addition to the covariates above, each ITS model included a linear monthly trend term for the preintervention period, an indicator variable for postmerger months (capturing any level change in the outcome postintervention), and a linear monthly trend term only for the postmerger months, which captures any change in slope of the outcome postintervention relative to preintervention.^12^ These terms allowed us to investigate whether the level and slope of the outcome changed postintervention. We estimated generalized linear models with the binomial family and logit link because our outcomes were binary.

A problem with the patient-level ITS is that it is difficult to both allow and control for the effects of autocorrelation. As a robustness check, the ITS models were repeated with aggregate monthly data. The presence of autocorrelation was checked for with the Durbin-Watson statistic, and the order of autocorrelation was detected by examining the plots of auto and partial correlation functions.^11^ As data management and analysis were performed with R version 4.03 (R Core Team) using the aggregate data, the ITS was reestimated correcting for autocorrelation using the R nlme library.^12^

## Results

The 122 348 patients in the premerger and 58 904 patients in the postmerger periods had a mean (SD) age of 55.5 (22.0) years the total sample of 181 252 patients included 112 191 women (61.9%), the payor mix was majority governmental (144 375 patients [79.7%]), and most admissions were emergent (121 469 patients [67.0%]) (Table 1). Patients from both time periods were largely clinically similar despite statistically significant differences, except that the case mix index score in the intervention period was higher. Medicare and Medicaid accounted for 80% of insurance coverage.

**Table 1.** Premerger and Postmerger Patient Characteristics at Time of Admission^a^

Characteristic	Patients, No. (%)		*P* value
	Premerger (n = 122 348)	Postmerger (n = 58 904)	
Age, mean (SD), y	54.9 (22.0)	55.4 (22.1)	<.001
Women	77 100 (63.0)	35 091 (62.2)	<.001
Men	45 248 (37.0)	21 351 (37.8)	
Payor			
Commercial	18 286 (14.9)	8686 (15.4)	<.001
Medicaid	48 756 (39.9)	23 620 (41.8)	
Medicare	49 548 (40.5)	22 451 (39.8)	
Other	2638 (2.2)	682 (1.2)	
Uninsured	3120 (2.6)	1003 (1.8)	
Case mix index, mean (SD)	1.36 (1.38)	1.47 (1.44)	<.001
Surgical DRG	33 621 (27.5)	16 813 (29.8)	<.001
Admission type			
Emergent	84 435 (69.0)	37 034 (65.6)	<.001
Elective	35 949 (29.4)	19 370 (34.3)	
Not available	1964 (1.6)	38 (<0.1)	

Abbreviation: DRG, diagnosis related group.

^a^ The premerger period was between September 1, 2010, and August 31, 2016; the postmerger period was September 1, 2016, through August 31, 2019.

**Figure 1.**
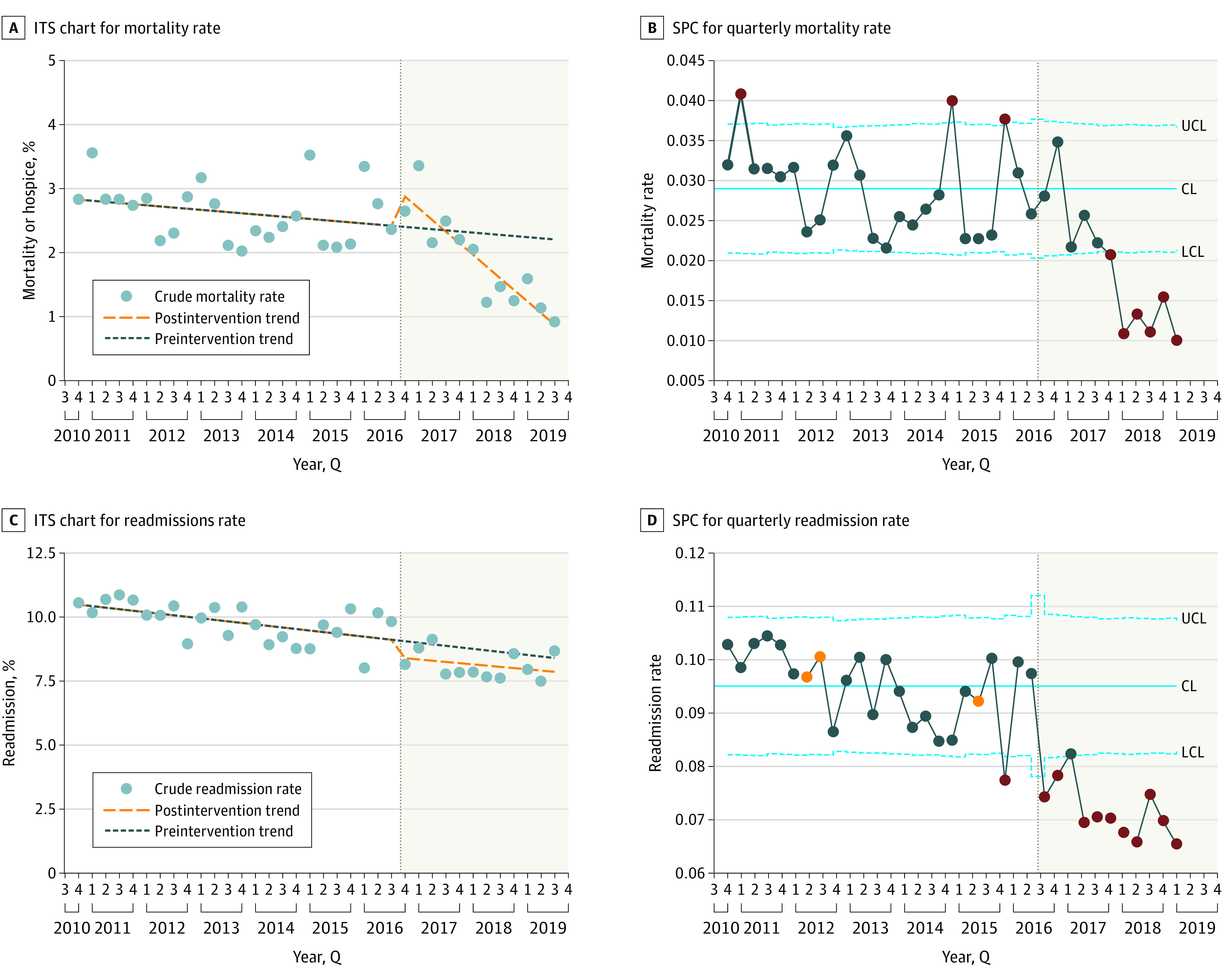
Interrupted Time Series (ITS) and Statistical Process Control (SPC) Charts of Mortality and Readmission Metrics Pre- and Postmerger For SPC charts, the upper and lower blue dotted confidence limits (CLs) denote bounds 3 SDs from the mean mortality rates indicated by the solid blue line. The eighth consecutive point below or above the mean and all subsequent ones are indicated in orange. Points outside the 3-SD range are indicated in red.

Unadjusted mortality declined from a mean of 2.6% in the premerger period to 1.9% postmerger, representing a 0.71% (95% CI, 0.57%-0.86%) absolute and 27% relative reduction (Figure 1). ITS analysis demonstrated a 1-time statistically significant mortality increase immediately after merger, followed by a statistically significant decrease postmerger that was greater than premerger trends (Table 2). Over the postmerger period, there was a 0.95% (95% CI, 0.83%-1.12%) absolute and 33% relative decrease in risk-adjusted mortality by the end of the 3-year intervention period (from an expected 1.96% without intervention based on the preintervention trend to 1.22% including postintervention effects, when holding the case mix index constant). An ITS model repeated with aggregate data demonstrated similar outcomes to patient-level ITS. Quarterly SPC analysis demonstrated special-cause variation in the postmerger period, notably including a run of 7 quarters below mean postmerger and 8 time points below 3 SDs (Figure 1). There was also special-cause variation in the premerger period, although notably with points above the mean.

**Table 2.** Interrupted Time Series of Mortality and Readmissions at Patient- and Aggregate-Level

Outcome	Preintervention trend		Postmerger step change		Postmerger trend change	
**Patient-level^a^**	**OR (95% CI)**	***P* value**	**OR 95% CI)**	**P value**	**OR (95% CI)**	***P* value**
Mortality	0.995 (0.993 to 0.997)	<.001	1.48 (1.28 to 1.71)	<.001	0.97 (0.96 to 0.97)	<.001
Readmission	0.998 (0.997 to 0.999)	.001	0.85 (0.79 to 0.92)	<.001	1.00 (0.996 to 1.003)	.83
**Aggregative-level **	**Change (95% CI), %**	***P* value**	**Change (95% CI), %**	***P* value**	**Change (95% CI), %**	***P* value**
Mortality	−0.01 (−0.04 to 0.01)	.25	0.78 (0.12 to 1.44)	.03	−0.17 (−0.24 to −0.09)	<.001
Mortality excluding first mo	−0.02 (−0.04 to 0.01)	.18	0.80 (0.13 to 1.47)	.03	−0.16 (−0.24 to −0.019)	<.001
Readmission	−0.004 (−0.06 to 0.06)	.89	−1.08 (−2.77 to 0.62)	.22	−0.04 (−0.23 to 0.15)	.66
Readmission excluding first mo	−0.06 (−0.09 to −0.03)	.001	−0.69 (−1.50 to 0.13)	.11	0.01 (−0.08 to 0.10)	.81

Abbreviation: OR, odds ratio.

^a^ Adjusted for age, sex, diagnosis related group weight, Elixhauser comorbidities, payer, surgical vs medical diagnosis related group weight and season.

Unadjusted readmission rates declined from an average of 9.5% premerger to 7.2% postmerger. SPC analysis demonstrated special-cause variation in adjusted readmission rates over time in the postmerger period, with a run of 11 quarters below the mean following the merger and all 11 points below 3 SDs (Figure 1). However, there was special-cause variation in the premerger period, and ITS analysis demonstrated no statistically significant difference between pre- and postmerger trends.

**Figure 2.**
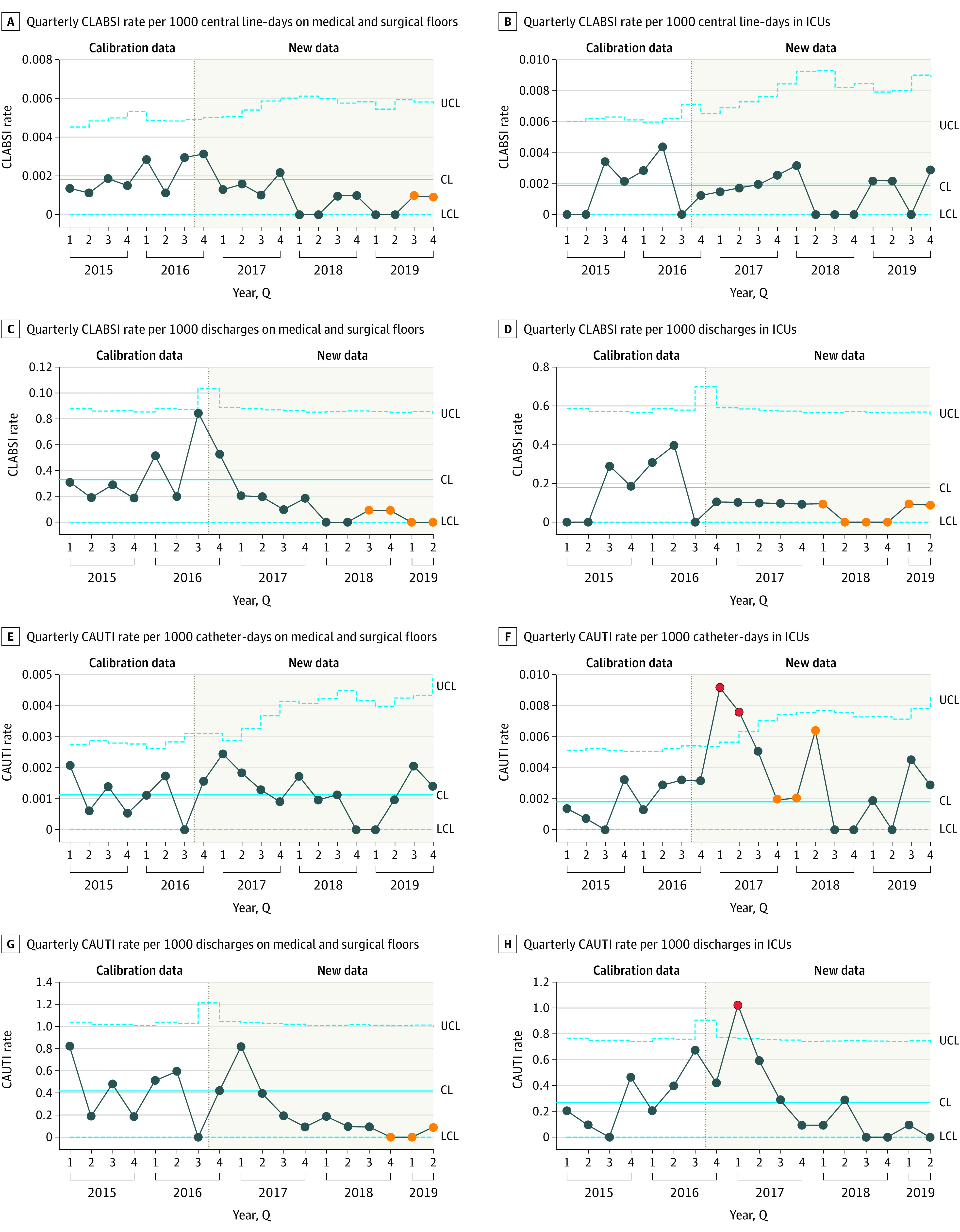
Statistical Process Control Charts of Central Line–Associated Bloodstream Infection (CLABSI) and Catheter-Associated Urinary Tract Infections (CAUTI) Metrics Pre- and Postmerger The upper and lower blue dotted confidence limits (UCL and LCL, respectively) denote bounds 3 SDs from the mean, indicated by the solid blue lines. The eighth consecutive point below or above the mean and all subsequent ones are indicated in orange. Points outside the 3-SD range are indicated in red.

The CLABSI rate postmerger showed prolonged special-cause variation for medical and surgical floors with both denominators; special-cause variation was observed only in CLABSI rate per 1000 discharges in intensive care units (Figure 2). CAUTI rates showed an early, transient increase in rates among intensive care unit patients, no meaningful change in the CAUTI rate per 1000 catheter days among medical and surgical floor patients, and a run of 9 quarters below the mean during the postmerger period in the CAUTI rate per 1000 discharges on medical and surgical floors. The hospital’s improvement in CAUTI is documented elsewhere.^13^

**Figure 3.**
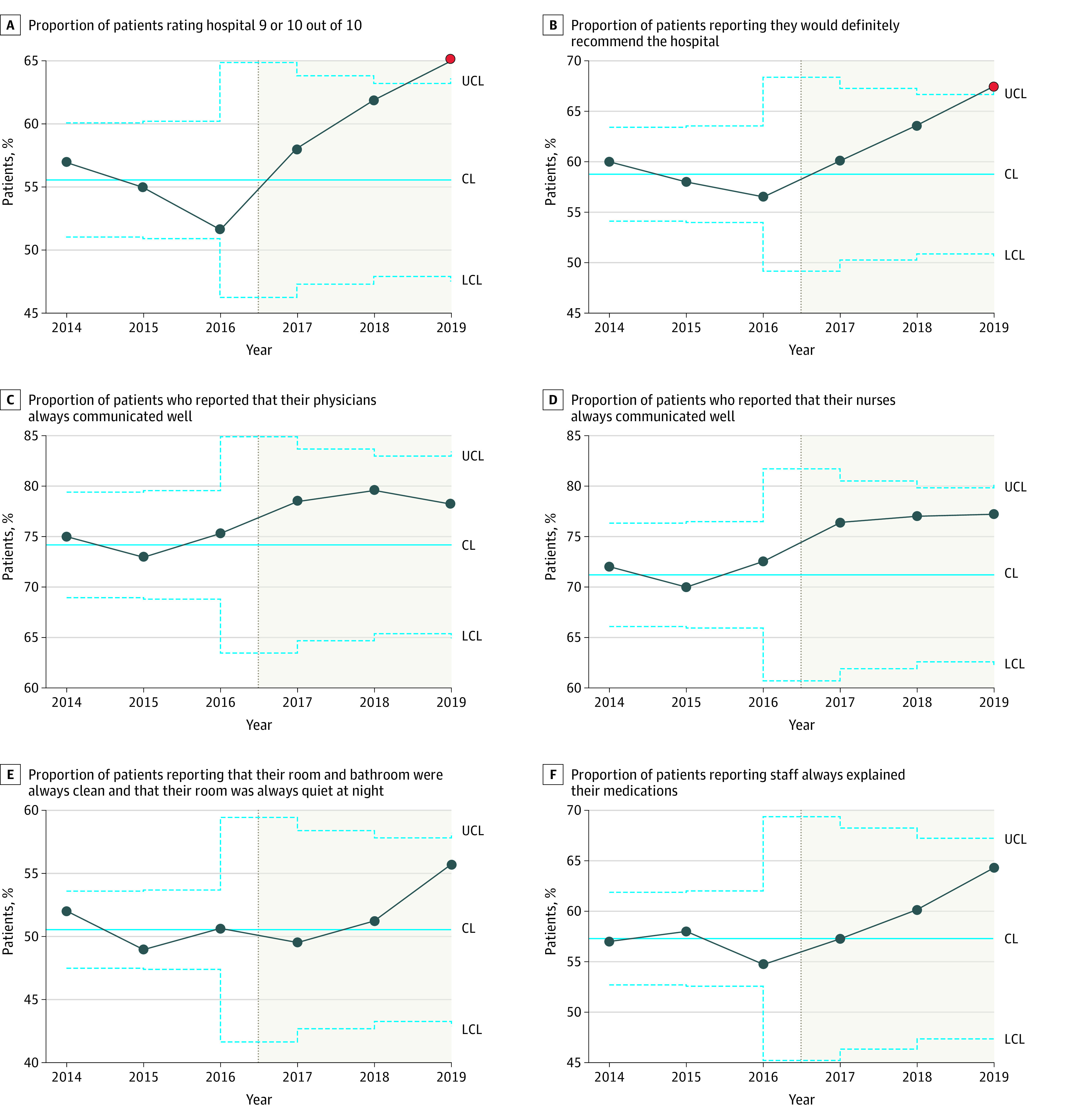
Statistical Process Control Charts of Patient Experience Metrics Pre- and Postmerger The upper and lower blue dotted confidence limits (UCL and LCL, respectively) denote the bounds 3 SDs from the mean, indicated by the solid blue line. Points outside the 3-SD range are indicated in red.

In the third year of the postmerger period, there was significant improvement in the proportion of 9 or 10 ratings in HCAHPS survey responses to the question, “Using any number from 0 to 10, where 0 is the worst hospital possible and 10 is the best hospital possible, what number would you use to rate this hospital during your stay?” (Figure 3). Similarly, in the third year of the postmerger period, there was significant improvement in the proportion of “definitely yes” responses to the question, “Would you recommend this hospital to your friends and family?” There was no change in the proportion of “always” answer to “During this hospital stay, how often did doctors listen carefully to you?” Improvement in the remaining questions assessed was not statistically significant.

## Discussion

We found that a full-integration approach to the acquisition of a resource-limited community hospital by an academic system was associated with improved clinical outcomes and patient experience, with the most substantial improvement in mortality with a 0.71% (95% CI, 0.57%-0.86%) absolute and 27% relative reduction in mortality rate across several ITS analyses. Special-cause variation was observed in SPC in the premerger period, with 3 time points above 3 SDs, although it is notable we observed a run of 7 quarters below the mean following the merger and 8 points that were below 3 SDs. There was also a single time point immediately after the merger well above the mean. It is common for quality outcomes to transiently worsen right after a significant systemic change. Similar results are seen with transitions to EHRs, where early increases in adverse events are later outweighed by long-term improvements.^14,15^ An eventual sustained improvement in mortality is likewise seen in our study. While SPC analysis showed an improvement in readmissions, ITS analysis confirmed this trend was also observed before the merger. There was statistically significant improvement in core patient experience measures and mixed improvement in HAC outcomes. In these ways, our approach and experience differed significantly from prior studies.

One study of New York hospitals found cardiac outcomes worsened and mortality increased after mergers.^16^ This and other studies have suggested hospital consolidations are usually intended to increase market share through negotiating power; procedures are often consolidated at a flagship hospital. Both may produce poorer quality outcomes at the acquired hospital. By contrast, NYULH operates in a hospital-dense environment and gained minimal market share from the acquisition. NYULHB had a largely governmental payer mix, conferring little to no negotiating power for NYULH. Service lines were expanded, not contracted, at NYULHB, which did not exist solely as a referral base. The goal of the merger was not revenue-driven; this uncommon full-integration approach was designed and executed to improve quality.

NYULH took an uncommon, value-driven approach in ensuring true integration, including a unified clinical and administrative governance structure supported by a robust electronic information system, including a common EHR and cost-accounting system. This focus on robust integration was balanced with identification of local opportunities, implementation of site-specific quality improvement interventions, and a systemwide adoption of some of these novel approaches. These innovations included nurse-driven and EHR-supported programs to reduce unnecessary urinary catheterization and, subsequently, CAUTIs; physician-led root cause analyses and occurrence reviews; and multidisciplinary workgroups to reduce the frequency and duration of hospitalization for high users of care.

In contrast, prior studies suggest a significant paucity of systems integration across most hospital acquisitions. Eickholt^9^ detailed why a fragmented approach to integration at 1 system (ie, a merger that lacked unified EHR or a single governance structure) led to an uncoordinated care delivery strategy and, subsequently, minimal change in outcomes. While that system did have shared clinical and financial targets, Eickholt observed an overemphasis on local autonomy at the expense of system strategy. In contrast, our system’s approach to consolidation sought to balance local circumstance with an integrated system strategy.

### Limitations

Our study had several limitations. First, while prior research reviewed the aggregate effect of mergers on quality, our study focused on a single hospital. Our system recently acquired another academic hospital, which may allow us to determine if our findings are replicable. Second, while we examined several quality measures, we could not assess all frequently studied quality metrics. Some secondary measures could not be assessed by ITS. Third, quality outcomes at the acquired hospital premerger were below average; the degree of success our merger produced may not be similarly achievable with other acquisitions. Notably, prior research found no improvement in mortality rates in aggregate, even with high-performing acquirers.^6^ Fourth, our study lacked a control hospital to account for secular trends. We did leverage both ITS and SPC to account for preintervention trends, a strength over a difference-in-differences design. ITS analysis does assume baseline trends would continue unchanged, which is also a limitation. Fifth, the authors are employees of the system; this relationship did allow us familiarity with details about merger activities and internal quality data. Sixth, as with many bundled interventions, we cannot disentangle which components of the approach, such as shared EHR or integrated clinical operations, were most significant.

## Conclusions

This study of a system merger with safety net hospital found that a full-integration approach to hospital consolidation was associated with improvement in quality outcomes. Despite evidence that mergers usually reduce quality, we found that strategic consolidations can be associated with substantially improved quality when performed effectively.
